# Characterization of the *Escherichia coli σ*^S^ core regulon by Chromatin Immunoprecipitation-sequencing (ChIP-seq) analysis

**DOI:** 10.1038/srep10469

**Published:** 2015-05-28

**Authors:** Clelia Peano, Johannes Wolf, Julien Demol, Elio Rossi, Luca Petiti, Gianluca De Bellis, Johannes Geiselmann, Thomas Egli, Stephan Lacour, Paolo Landini

**Affiliations:** 1 Institute of Biomedical Technologies, National Research Council (ITB-CNR), Segrate (MI), Italy; 2 EAWAG, Swiss Federal Institute for Environmental Science and Technology, Dübendorf, Switzerland; 3 Lab. Adaptation et Pathogénie des Micro-organismes (LAPM), Univ. Grenoble Alpes, F-38000 Grenoble, France; 4 UMR 5163, Centre National de Recherche Scientifique (CNRS), Grenoble, France; 5 Department of Biosciences, Università degli Studi di Milano, Milan, Italy

## Abstract

In bacteria, selective promoter recognition by RNA polymerase is achieved by its association with σ factors, accessory subunits able to direct RNA polymerase “core enzyme” (E) to different promoter sequences. Using Chromatin Immunoprecipitation-sequencing (ChIP-seq), we searched for promoters bound by the σ^S^-associated RNA polymerase form (Eσ^S^) during transition from exponential to stationary phase. We identified 63 binding sites for Eσ^S^ overlapping known or putative promoters, often located upstream of genes (encoding either ORFs or non-coding RNAs) showing at least some degree of dependence on the σ^S^-encoding *rpoS* gene. Eσ^S^ binding did not always correlate with an increase in transcription level, suggesting that, at some σ^S^-dependent promoters, Eσ^S^ might remain poised in a pre-initiation state upon binding. A large fraction of Eσ^S^-binding sites corresponded to promoters recognized by RNA polymerase associated with σ^70^ or other σ factors, suggesting a considerable overlap in promoter recognition between different forms of RNA polymerase. In particular, Eσ^S^ appears to contribute significantly to transcription of genes encoding proteins involved in LPS biosynthesis and in cell surface composition. Finally, our results highlight a direct role of Eσ^S^ in the regulation of non coding RNAs, such as OmrA/B, RyeA/B and SibC.

Bacteria are constantly exposed to changes and fluctuations in their environment, to which they can adapt by reprogramming their gene expression through various mechanisms, including use of alternative σ factors. σ factors are accessory subunits of bacterial RNA polymerase that associate, in a 1:1 stoichiometric ratio, to the core enzyme (E), *i.e.*, the multi-subunit complex responsible for RNA polymerase catalytic activity. Binding to any of the different alternative σ factors creates different RNA polymerase holoenzymes (Eσ), proficient in specific promoter recognition and transcription initiation. After the process of transcription initiation has taken place, the σ factor dissociates from the holoenzyme, and the core enzyme carries out transcription elongation^1^. The number of σ factors varies considerably among bacteria: seven σ factors are known to be present in *Escherichia coli*, including σ^70^ (or σ^D^), the “housekeeping” σ factor devoted to transcription of a large part of the genome and of most essential genes. In contrast, alternative σ factors are responsible for the transcription of smaller subsets of genes, fulfilling specific roles or belonging to defined functional groups^2^. One alternative σ factor, σ^S^, strongly affects cell survival during stress conditions, such as starvation, oxidative stress, and exposure to either low or high pH, and controls expression of virulence factors in several pathogens^3^. For its important role in response to cellular stresses, σ^S^ is considered the master regulator of the so-called “general stress response” and, consistently, it is induced in response to any stressful event leading to reduction in specific growth rate^4^,^5^.

Interestingly, σ^S^ and σ^70^ appear to recognize very similar promoter sequences^6^. Consequently, several promoters are recognized with similar efficiency by both Eσ^S^ and Eσ^70^ *in vitro*^7^, and their preferential recognition by either form of RNA polymerase *in vivo* is mediated by accessory regulatory proteins^6^. Selective promoter recognition by either σ^70^ or σ^S^ can be achieved by deviations from a common consensus sequence^6^,^8^ which confer specificity for either σ factor: for instance the presence of a C nucleotide (−13C) immediately upstream of the −10 promoter element is a known determinant for σ^S^ binding and it is a common feature in σ^S^-dependent promoters^9^. In a previous work, we set out to determine which promoters are preferentially bound *in vitro* by either Eσ^70^ or Eσ^S^ by run-off transcription microarray (ROMA); we confirmed the importance of sequence elements important for promoter recognition by σ^S^, such as the presence of C residues at positions -13 and -12 C element, and suggested that an A/T-rich discriminator region would favour transcription initiation by Eσ^S^ *in vitro*^10^.

In this work, we used Chromatin-Immunoprecipitation-sequencing (ChIP-seq) to identify promoters bound by Eσ^S^ at early stationary phase, *i.e.*, at a moment in which σ^S^ accumulates inside the bacterial cell. Our results led to identification of novel σ^S^-dependent genes, and provided insight on regulation of non-coding RNAs by σ^S^. We could also show that a significant subset of Eσ^S^-bound promoters controls genes whose expression is σ^S^-independent, suggesting considerable overlap in promoter recognition by different σ factors.

## Results

### MG1655-*rpoS*_His6_ construction and σ^S^_-His6_ immunoprecipitation

**Figure 1:**
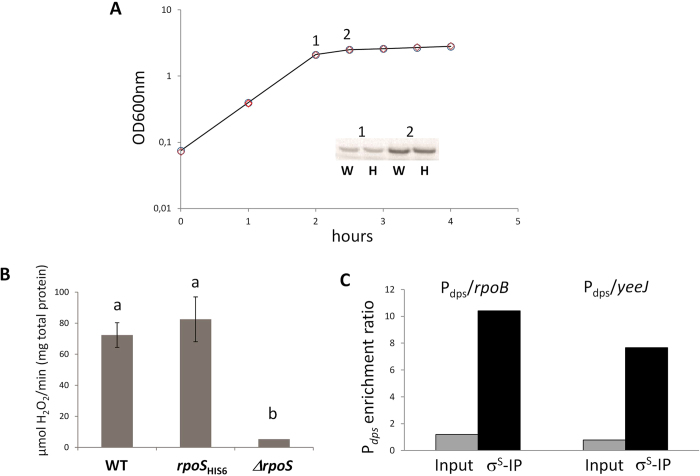
Characterization of the MG1655-*rpoS*_His6_ mutant. **A**. Growth curves in LB medium of MG1655 (circles) and MG1655-*rpoS*_His6_ (diamonds) strains. Intracellular amount of σ^S^ (for MG1655) and σ^S^-_His6_ (for MG1655-*rpoS*_His6_) as determined by western blot at the onset of stationary phase (points 1 and 2 in the graph) are shown in the inset. **B**. HPII catalase specific activity in MG1655, MG1655-*rpoS*_His6_ and in the MG1655Δ*rpoS* strains. Values from three independent experiments were analyzed by ANOVA; the letters indicate samples showing statistically significant differences. **C**. Determination of relative abundance of the *dps* promoter region in the Immunoprecipitated (IP) versus the Input sample by RT-PCR. Data are the average of two repeats with identical results.

Since no anti-σ^S^ antibodies suitable for immunoprecipitation were available at the time of this study, we decided to utilize anti-6xHis-tag antibodies targeting a histidine-tagged σ^S^ protein (σ^S^_-His6_). In order to study promoter binding by σ^S^_-His6_ without perturbing σ^S^ physiological levels or *rpoS* gene expression, we constructed a strain carrying a chromosomal *rpoS*_His6_ allele, *i.e.*, an otherwise wild type *rpoS* allele with 6 codons for histidine at its 3` end, as described in Materials and Methods. We verified the effects of the *rpoS* allele replacement on specific growth rate (Fig. 1A) and checked the relative amounts of both the wild type and the σ^S^_-His6_ proteins at the onset of stationary phase by Western blot, using an anti-σ^S^ antibody (Fig. 1A, inset). A Western blot with the anti-6xHis antibody confirmed that the MG1655-*rpoS*_His6_ strain did indeed produce a 6xHis-tagged σ^S^ protein (data not shown). No differences were detected in either specific growth rate or intracellular σ^S^ amounts in the two strains (Fig. 1A). Western blot analysis clearly showed that, as expected, the amount of σ^S^ (or σ^S^_-His6_) increased significantly at the end of the exponential phase, (compare points 1 and 2): at this point, bacterial cells were growing at a specific growth rate of 0.32 (±0.02) h^−1^. Cells were collected at the growth stage corresponding to point 2 in Fig. 1A in all subsequent experiments.

To verify whether the C-terminal histidine tag might affect σ^S^ activity *in vivo*, we tested the activity of HPII catalase, encoded by the *rpoS*-dependent *katE* gene and a marker for *rpoS* functionality^11^. No statistically significant difference in HPII specific activity was detected between MG1655 and MG1655-*rpoS*_His6_, while, in contrast, HPII catalase specific activity was almost totally abolished in an *rpoS* null mutant strain, as expected (Fig. 1B). These results indicate that introduction of the 6xHis-tag in the σ^S^ protein does not affect its abundance, physiological regulation and activity. Thus, we performed protein-DNA co-immunoprecipitation experiments in the MG1655-*rpoS*_His6_ strain, using anti-6xHis antibodies. As a quality control of the co-immunoprecipitation experiment, we verified the enrichment of a known binding site for Eσ^S^ in the immunoprecipitated samples compared to sonicated DNA (Input sample). To this purpose, we performed qRT-PCR experiments comparing the relative abundance of the promoter region of the σ^S^-dependent *dps* gene (P*dps*) to coding sequences within the *rpoB* and the *yeeJ* genes. Both the P*dps*/*rpoB* and P*dps*/*yeeJ* ratios approached 1 in the Input sample, while being 10-fold higher in the σ^S^_-His6_ immunoprecipitation sample (σ^s^-IP; Fig. 1C), thus suggesting strong enrichment in Eσ^S^ binding sites by the immunoprecipitation procedure.

### Chromatin immunoprecipitation-sequencing (ChIP-seq)

**Figure 2:**
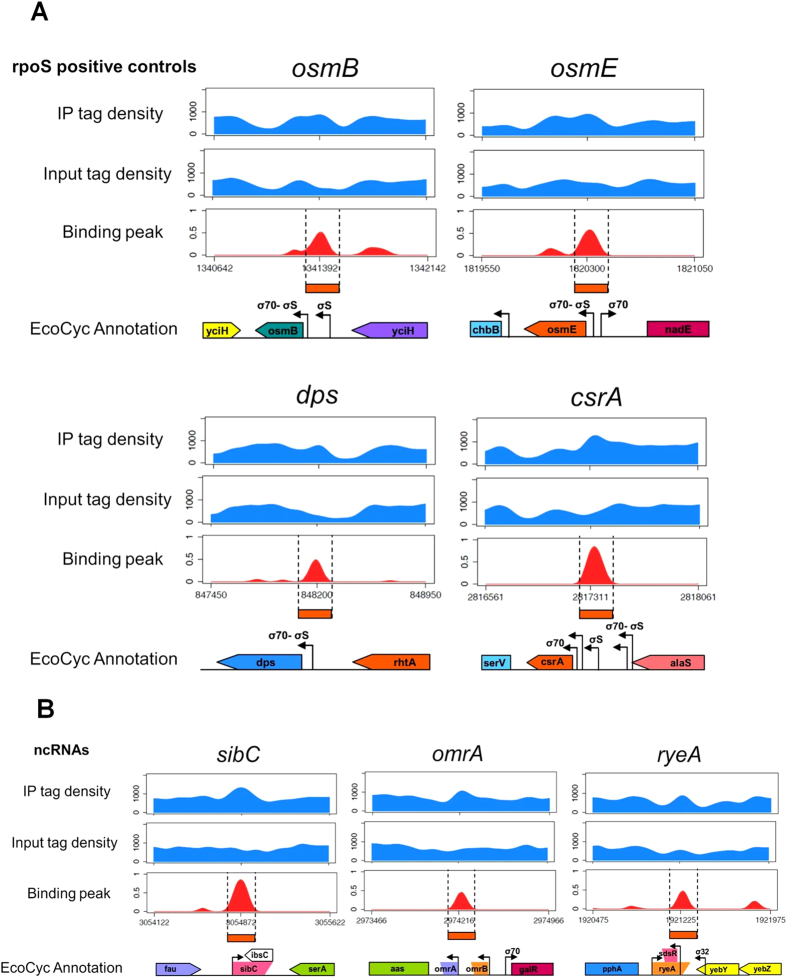
Visualization through IGV of the binding peaks obtained from CisGenome analysis. The blue profiles show the IP and Input tag density profiles for the known *rpoS*-dependent genes *osmB*, *dps*, *osmE* and *csrA* (**A**) and for the loci associated to the non-coding RNAs *sibC*/*ibsC*, *ryeA*/*ryeB*, and *omrA*/*omrB* (**B**). The red profiles show the log_2_ signal to control enrichment estimates values obtained using spp (peaks) for the same genes and non-coding RNAs. Values on X axis are the genomic coordinates of the peaks; a representation of the corresponding gene/intergenic regions taken from Ecocyc (ecocyc.org) is shown.

Two replicates of the Input sample (MG1655-*rpoS*_His6_ chromosomal DNA) and of the σ^S^-IP sample (σ^S^_-His6_ immunoprecipitated DNA) were used to prepare sequencing libraries. The libraries were sequenced into 4 separate lanes of the same GAIIx run. We obtained more than 50 million mapping reads for both the input samples (corresponding to a sequencing depth of 543-fold the *E. coli* genome); for the first and the second IP samples, more than 26 and 32 million mapping reads were obtained, respectively. Identification of the DNA regions more represented in the σ^s^-IP sample, corresponding to potential binding sites for Eσ^S^, was carried out using the CisGenome software^12^, which yielded 78 “peaks”, *i.e.*, regions of the genome significantly enriched (pval ≤ 0.01) in the σ^s^-IP sample as compared to the Input sample. Almost all peaks detected (72/78) corresponded to DNA regions ≤400 bp-long or slightly larger, consistent with the DNA fragment sizes obtained after DNA sonication (see Materials and Methods, “σ^S^_-His6_ *immunoprecipitation*”). Three enriched regions were slightly larger in size (500-700 bp), while only three regions had sizes larger than 1kbp (1049, 1199 and 3149 bp, respectively). The last one encompassed a DNA region including five different ORFs and several non-coding and regulatory elements, making it impossible to identify a putative binding site for Eσ^S^; thus, this DNA fragment was excluded from further analysis and is listed, together with intragenic peaks, in Supplementary Table S2 (see below). On the contrary, the two peaks just over 1 kbp overlapped a single known promoter region, and were thus included in the Eσ^S^ binding site analysis shown in Table 1. The visualization through Integrative Genome Viewer (IGV) of representative σ^S^ binding peaks obtained from the CisGenome analysis is shown in Fig. 2: significantly enriched genomic regions (*i.e.*, peaks) are reported for the known *rpoS*-dependent genes *osmB*, *dps*, *osmE* and *csrA* (Fig. 2A) and for loci associated to the small RNAs *sibC/ibsC*, *ryeA/ryeB*, and *omrA/omrB* (Fig. 2B; see also section “*Regulation of non-coding RNA by Eσ*^*S*^”).

The large majority (63 out of 78) of the σ^S^-IP peaks was located immediately upstream of coding sequences or known regulatory RNAs, consistent with σ^S^ binding to promoter regions. Out of these 63 peaks, 61 were located in intergenic regions, while two peaks lie within the *stfR* and *wbbH* ORFs, but upstream, respectively, of the *tfaS* and *wbbI* genes, suggesting that they might define internal promoters within operons. The remaining peaks fell into intragenic regions at considerable distance from other ORFs (listed in Supplementary Table S2). Although it is possible that some of these peaks might define *bona fide* Eσ^S^ binding sites (*e.g.*, promoters for yet unknown antisense RNAs), they were not considered for further characterization within this study. However, even assuming that all the intragenic peaks are artefacts of ChIP-seq, the resulting percentage of false positives (19%) would still be lower than what reported for similar studies^13^.

50 out of the 63 peaks corresponding to known or putative promoter regions could unequivocally be attributed to one specific gene, based on the DNA sequence covered by the peak, the direction of transcription of the neighbouring genes, the distance to the nearest ORFs and, when available, the presence of an experimentally determined transcription start site within the boundaries of the peak. Of the 50 genes unequivocally identified, 27 had been shown to be at least partially *rpoS*-dependent in previous reports, as listed in Table 1. In contrast, 13 peaks, listed in Table 2, lie in intergenic regions between divergently transcribed genes or operons and could not be assigned to a specific gene. However, we often found that one of the two divergent genes (or even both, as for the *dsrB-yodD* intergenic region, Table 2) had previously been described as *rpoS*-dependent, thus suggesting that Eσ^S^ binding was due the presence of an *rpoS*-dependent promoter within the intergenic region. As an example, we assigned the putative Eσ^S^ binding site in the *osmE-nadE* intergenic region to *osmE*, since its promoter is σ^S^-dependent^14^,^15^,^16^ (Fig. 2 and Table 2).

Altogether, the peaks identified in the ChIP-seq experiment overlapped with the promoters of 36 genes that had been shown to be at least partially *rpoS*-dependent (highlighted in Tables 1 and 2). Stress-related genes defined the most represented functional category in our ChIP-seq analysis (see Tables 1, 2, [Table t2]), in agreement with the role of σ^S^ as master regulator of the general stress response. Interestingly, binding sites for Eσ^S^ were also found upstream of several genes involved in cell envelope structure (*erfK*, *lpp*, *ynhG*) and lipopolysaccharide (LPS) biogenesis (*lpxC*, *wbbH*, *wbbI*), suggesting that Eσ^S^ might be important for the expression of cell surface-related genes in response to growth cessation.

The majority of the intergenic regions not linked to *rpoS*-dependent genes included known or putative promoters recognized by Eσ^70^, in agreement with previous results indicating extensive cross-recognition between Eσ^S^ and Eσ^70^ regulons^7^,^9^. Interestingly, however, several promoters are also recognized by other alternative σ factors, namely σ^E^ (*ytfJ* and *lpxP*) and σ^H^ (*hepA*, *sdaA*, *raiA* and *rpmE*) (Tables 1, 2, [Table t2]).

### *In vivo* expression of genes identified by ChIP-seq analysis

The results of our ChIP-seq experiments seem to indicate that a large percentage of Eσ^S^-binding sites are associated with promoters directing transcription of *rpoS*-independent genes. Alternatively, regulation of these genes by σ^S^ might have been overlooked in previous investigations of the *rpoS* regulon, mostly carried out as whole genome transcription analysis comparing an *rpoS* mutant to its parental strain^14^,^15^,^16^,^17^,^18^,^19^. In order to elucidate the functional role of the Eσ^S^-binding sites, we measured relative expression of 10 genes whose promoters, according to our ChIP-seq results, are recognized by Eσ^S^, by performing qRT-PCR experiments comparing *E. coli* MG1655 to its otherwise isogenic *rpoS* mutant. As control genes in the qRT-PCR experiment, we chose 4 genes previously proposed to be *rpoS*-dependent: *dps*, *ycgB*, *rssA* and *bsmA*^15^,^16^,^20^. The remaining 6 genes, never previously shown to be *rpoS*-dependent, were selected based either on their function or on promoter features: *lpp* encodes Braun lipoprotein, which bridges the outer membrane to peptidoglycan and is extremely abundant in *E. coli*^21^; *ssrA* is a transfer-messenger RNA (tmRNA)-encoding gene; *uxaB* is involved in galacturonate metabolism; *ybiI* is a gene of unknown function whose promoter had been indicated as putative Eσ^S^-dependent through bioinformatics prediction^22^; *ydbK* is an oxidative stress-related gene^23^; *ygjR*, like *ybiI*, is an unknown function gene with a known transcription start site^24^, whose putative −10 region shows some features typical of Eσ^S^-dependent promoters, such as the −13C.

**Figure 3:**
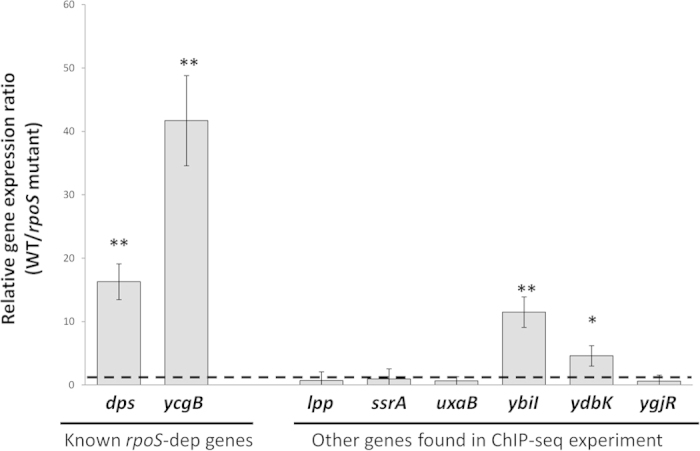
RT-PCR analysis. The Relative expression ratio between WT and *rpoS* mutant indicated in the graph are the average of at least four experiments (two repeats, each performed on duplicate samples, from two independent RNA extractions), and standard deviations are shown. The asterisks denote significant differences (*=*p* < 0.05; **= *p* < 0.01 Tukey multigroup analysis). The dashed line indicates a WT/*rpoS* mutant expression ratio=1.

Results of the qRT-PCR experiments (Fig. 3) could demonstrate *rpoS*-dependent gene expression for *dps*, *ycgB*, *ybiI* and *ydbK*, suggesting that the latter two are yet unidentified members of the *rpoS* regulon. In contrast, the expression of the remaining genes was not affected by the lack of a functional *rpoS* gene, at least in the conditions tested. To further investigate whether these genes showed any kind of dependence on σ^S^, we tested their expression levels in a *rpoS*-overexpressing strain (MG1655/pBADrpoS) grown to early stationary phase in LB medium supplemented with 0.1% arabinose. Although intracellular σ^S^ amounts were almost 10-fold higher in the pBADrpoS-bearing strains compared to MG1655, no significant changes in relative expression levels were detected for any of the genes tested (data not shown).

### *In vitro* Eσ^S^-promoter interactions

**Figure 4:**
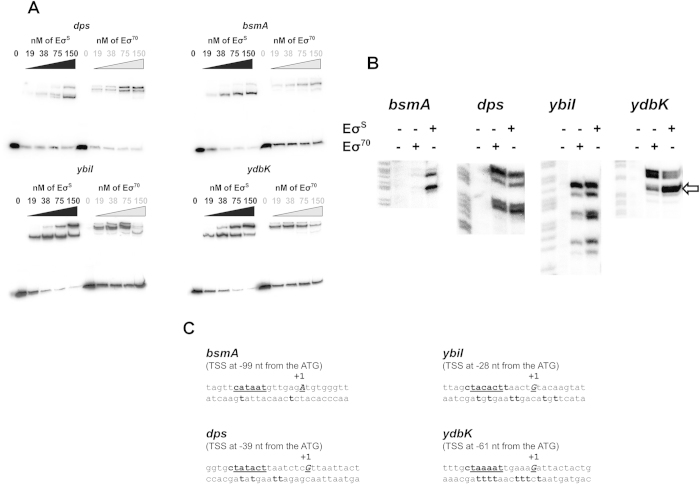
Eσ^S^-promoter interactions *in vitro*. **A.** Gel retardation assays performed in K-glutamate buffer with heparin challenge. **B.** KMnO_4_ reactivity assays: both Eσ^S^ and Eσ^70^ forms of RNA polymerase were tested at 50 nM. For each panel, the first lane is a molecular weight marker obtained as a G+A sequencing reaction of the DNA fragment. **C.** Sequence of the newly identified *bsmA*, *ybiI* and *ydbK* promoters. Sequences are given from position −17 to +10 according to the transcription start site (TSS) labelled “+1” and indicated in bold. The −10 promoter element is underlined. KMnO_4_-reactive thymidine residues in the template strand (labelled with ^32^P) reactive in the KMnO_4_ assays are indicated in bold.

Results of the ChIP-seq and qRT-PCR experiments failed to show strong correlation between Eσ^S^ promoter binding and Eσ^S^-dependent transcription, even for genes previously described as *rpoS*-dependent, such as *rssA* and *bsmA* (Fig. 3). In order to confirm ChIP-seq results, we studied Eσ^S^-promoter interactions *in vitro*, by comparing Eσ^S^ and Eσ^70^ for their ability to bind and to promote open complex formation at a subset of the promoters studied in qRT-PCR experiments. We selected the promoter regions of the two newly identified *rpoS*-dependent genes, *ybiI* and *ydbK*, together with the promoters of the known *rpoS*-dependent *dps* and *bsmA* genes, which, however, showed different behaviour in our qRT-PCR experiments. Firstly, we performed GMSA with either Eσ^S^ or Eσ^70^, in the presence of heparin to select for open complexes, on regulatory DNA fragments (extending from 250 bp upstream to 30 bp downstream of the start codon). Eσ^S^ was clearly more efficient than Eσ^70^ in promoting open complex formation at the *ybiI*, *ydbK* and *bsmA* promoters (compare amounts of unbound DNA probes, Fig. 4A), while both forms of RNA polymerase showed similar proficiency in open complex formation at the *dps* promoter, despite its strong Eσ^S^-dependence *in vivo* (Fig. 3; 8,16). As a negative control for binding by Eσ^S^, we performed GMSA experiments on the strictly Eσ^70^-dependent *crl* promoter, which clearly showed preferential binding by Eσ^70^ (Supplementary Fig. S1).

To further investigate promoter DNA-RNA polymerase interaction, and to map the exact location of the -10 promoter elements for *ybiI*, *ydbK* and *bsmA*, we performed KMnO_4_ reactivity assays (Fig. 4B). Treatment with permanganate oxidizes thymidine residues in single-stranded DNA, allowing us to identify precisely the location of open complexes. As expected, no open complex formation by Eσ^S^ was detected at the Eσ^70^-dependent *crl* promoter (Supplementary Fig. 1). In contrast, open complex formation at the *bsmA* promoter was only observed in the presence of Eσ^S^, consistent with GMSA results and confirming specific recognition by Eσ^S^ at this promoter . Similarly, at the *ybiI* promoter, binding by Eσ^S^ resulted in much stronger reactivity than Eσ^70^, indicating more efficient open complex formation. A more complex picture emerged from KMnO_4_ experiments at the *ydbK* promoter, which showed that both Eσ^S^ and Eσ^70^ can recognize a promoter located, in agreement with bioinformatics predictions^22^, at ca. 70 nucleotides upstream of the *ydbK* ORF. However, subtle changes can be observed in the pattern of KMnO_4_ reactivity induced by the two RNA polymerase-promoter complexes, with binding by Eσ^S^ resulting in higher reactivity in the T residues at positions −4 to −2 (marked by an arrow in Fig. 4B). Taken together with GMSA results, this observation suggests that, at the *ydbK* promoter, Eσ^S^ might trigger formation of an open complex more resistant to heparin challenge and possibly more proficient in transcription initiation. Finally, at the *dps* promoter, both Eσ^S^ and Eσ^70^ induced open complex formation with equal efficiency, indicating lack of preferential recognition by either form of RNA polymerase *in vitro*.

### Regulation of non-coding RNAs by Eσ^S^

**Figure 5:**
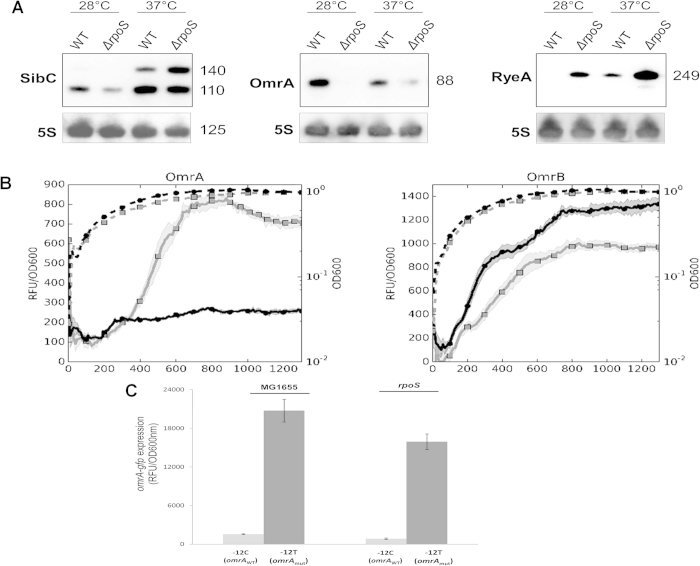
Regulation of small non-coding RNAs by σ^S^. **A.** Northern blot hybridization. RNA were extracted at the onset of stationary phase (OD600nm of 3) from bacteria grown in LB at either 28 °C or 37 °C and probed for SibC, OmrA, and RyeA transcript levels (left to right). Numbers on the right side of each panel indicate the size of the respective ncRNA. The gels were probed for the genes of interest, then the probe was removed by washing and the gels were re-probed for 5S RNA, which was used as internal control. **B.** Relative fluorescence of transcriptional fusions of the *omrA* and *omrB* promoters to the GFP reporter gene. The promoter activity (solid line) is expressed as ratio between the fluorescence and the absorbance of the culture (dashed line) after background correction (RFU/OD600 nm). **C.** Effects of the substitution of the −12C to a T nucleotide in the *omrA* promoter region. Data were taken from overnight cultures and are the average of four independent experiments.

Results of ChIP-seq analysis indicate that three Eσ^S^ binding sites are positioned in the proximity of genes encoding regulatory RNAs. A putative Eσ^S^ binding site was identified upstream of the 88 nt-long regulatory RNA *omrA*, which controls expression of genes involved in flagellar motility, iron uptake, adhesion factors and various outer membrane proteins^25^. The *omrA* gene lies next to *omrB*, which codes for a highly similar small RNA and also regulates some of the targets for *omrA*^25^,^26^. The other two Eσ^S^ binding sites were found in proximity of two complex loci: the *ryeA/ryeB* locus, which includes two small RNAs overlapping in antisense directions^27^, and the *sibC/ibsC* locus, in which a non coding RNA (*sibC*) overlaps a small ORF, *ibsC*, reading in the opposite direction, and encoding a toxic peptide^28^. The location and extension of the three ChIP-seq peaks suggest that Eσ^S^ might bind the promoter regions of *omrA* (but not *omrB*), and of *ryeB* and *sibC*, rather than *ryeA* and *ibsC* (Fig. 2B), consistent with recent observations that *omrA* and *ryeB* are *rpoS*-dependent in *Salmonella enterica*^29^,^30^. To confirm this result, we performed northern blots comparing small RNA levels in the wild type versus the *rpoS* mutant strain of *E. coli* (Fig. 5). In addition to standard growth conditions (LB medium at 37 °C), we also carried out northern blot experiments at 28 °C, since low growth temperature favors σ^S^ accumulation and positively affects stability of some small RNA^31^. Due to difficulties in obtaining a clean result with a probe for RyeB, we measured the relative amounts of RyeA, which upon pairing with RyeB, is degraded in an RNaseIII-dependent fashion and shows therefore transcript levels inversely proportional to *ryeB*^27^,^29^. Inactivation of the *rpoS* gene almost abolished *omrA* transcription, while strongly increasing RyeA transcript levels (Fig. 5A), consistent with *rpoS*-dependence of transcription of the *omrA* and *ryeB* genes. Interestingly, the OmrA and RyeA transcripts also displayed opposite temperature-dependence, with OmrA being more expressed at 28 °C and RyeA at 37 °C. As further confirmation that *rpoS*-dependent regulation specifically targets *omrA*, but not *omrB*, we performed *gfp* reporter assays. Reporter genes experiments clearly showed very different effects of *rpoS* inactivation on transcription of the two genes, with *omrA* showing almost complete *rpoS*-dependence, while *omrB* expression was actually slightly increased in the *rpoS* mutant background (Fig. 5B). Interestingly, the first nucleotide of the −10 region of *omrA* is a −12C (Supplementary Table S3), a feature favouring specific promoter opening by Eσ^S^ but not by Eσ^70^ 32, while at the *omrB* promoter, such a selective determinant is replaced by a canonical −12T for Eσ^70^ and might explain lack of preferential binding by Eσ^S^. Substitution of the −12C nucleotide by a −12T in the *omrA* −10 promoter element increases promoter strength by more than 10-fold and almost completely overcomes its dependence on *rpoS* (Fig. 5C), suggesting that the −12C act as a determinant for Eσ^S^ specificity in the *omrA* promoter. A more complex picture emerged from analysis of the SibC transcript, which, like RyeA, showed increased expression at 37 °C than at 28 °C. At the latter temperature, SibC was transcribed in an *rpoS*-dependent manner; however, the effect of the *rpoS* mutation was reversed at 37 °C, possibly suggesting additional regulatory mechanism affecting SibC expression at this temperature (Fig. 5A). The complexity of SibC regulation is also suggested by the presence of two transcripts, either due to the presence of multiple promoters or to RNA processing as already described^28^.

### Sequence analysis of σ^S^-bound promoters

**Figure 6:**
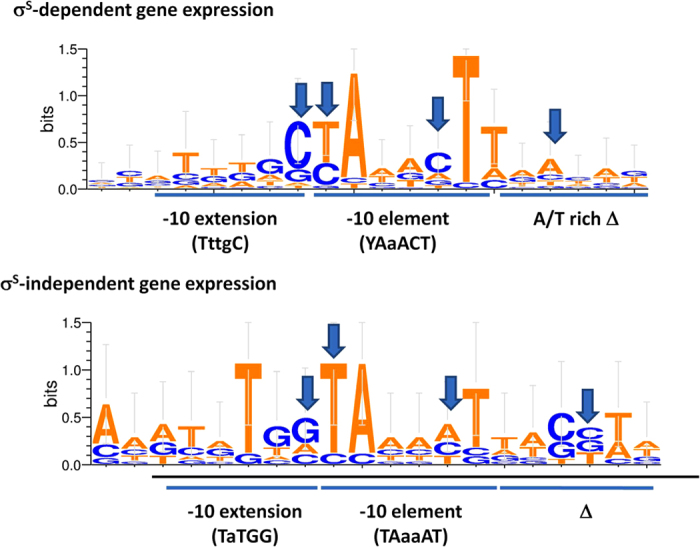
Promoter sequence alignment. Weblogo 3 (http://weblogo.threeplusone.com/) representation of the sequence alignments for experimentally identified promoters located within Eσ^S^ binding sites. −10 regions of either σ^S^-dependent (top panel) or σ^S^-independent genes (bottom panel) were aligned setting the first nucleotide of the −10 hexamer as −12 position. Promoter sequences are reported in Table S3.

In order to assess the importance of σ^S^-specific promoter determinants for binding by σ^S^, we analyzed the sequences of the experimentally determined promoters controlling genes identified in the ChIP-seq experiments (30 promoters, listed in Supplementary Table S3). The promoters were divided in two subsets: the ones directing transcription of genes reported to show some level of dependence on σ^S^ (21 promoters) and those controlling genes whose expression is not affected by lack of a functional *rpoS* gene (9 promoters). In good agreement with the previously proposed consensus for σ^S^ 4,8,10,16, −10 region alignment of σ^S^-dependent genes (from −20 to +1, Fig. 6) suggests that their consensus sequence in the −17 to −6 region would be TNTGCYAAACTT, where N is any nucleotide and Y is a pyrimidine and W is either A or T (Fig. 6); in addition, promoters of σ^S^-dependent genes are characterized by an A/T-rich discriminator region. Promoters of σ^S^-independent genes lack conservation of the C residues at positions −13, −12, and −8, reduced frequency of a T at position −6, and display a discriminator region richer in G/C (Fig. 6). Alignment of the −35 regions of σ^S^-bound promoters (listed in Supplementary Table S4) highlighted some conservation of the σ^70^ consensus sequence, TTGACA, in the promoters of genes whose expression is independent of σ^S^ ; in contrast, in the promoters of σ^S^-dependent genes, the −35 region showed a weakly conserved sequence, GCTGACAAA, with some resemblance to the −35 promoter element for σ^70^ (Supplementary Fig. S2). It remains to be understood whether this sequence might play any role in σ^S^ –promoter interactions.

## Discussion

In this work, we used a ChIP-seq approach in order to identify promoters bound by Eσ^S^ during the early stationary phase, in which σ^S^ concentrations surge in the bacterial cell (Fig. 1A). The experimental conditions used in this work were chosen in order to identify genes directly regulated by σ^S^ that are induced in response to transition into stationary-phase. Indeed, we only detected 63 promoter regions bound by Eσ^S^ (Tables 1, 2, [Table t2]); this number only represents a fraction of the σ^S^-bound promoters previously identified either by microarray or by ChIP-on-chip analysis^14^,^19^,^33^, which, however, were performed under a variety of different growth conditions and include genes subject to complex regulation and only indirectly regulated by σ^S^. Out of the 63 promoters identified in our study, 38 (60%) control transcription of genes regulated by the σ^S^-encoding *rpoS* gene (Tables 1, 2, [Table t2] and references within). Two of these, *ybiI* and *ydbK*, had not yet been identified as part of the *rpoS* regulon, and we confirmed their preferential recognition by Eσ^S^ via *in vitro* binding and open complex formation experiments (Fig. 4). However, a large percentage of σ^S^-bound promoters control genes whose expression is not affected by the presence of this factor (see Tables 1, 2, [Table t2], Fig. 3), suggesting that these promoters are recognized with similar efficiency by σ^S^ and other σ factors, mostly σ^70^. This result is consistent with the notion that σ^S^ does not only serve to promote expression of its own regulon, but it can also contribute to transcription of constitutively expressed genes. Promoter sequence comparison between *bona fide* σ^S^-dependent genes and those not showing altered expression in an *rpoS* mutant highlighted the importance of the promoter elements associated with selective recognition by σ^S^ (Fig. 6). At least some σ^S^-specific determinants might be more important for preventing recognition by σ^70^ *in vivo* rather than increasing binding affinity or promoter opening by σ^S^, such as the presence of a C rather than a T as first nucleotide of the −10 hexamer, as is the case at the *omrA* promoter (Fig. 5C). Although the mechanisms of regulation by σ^S^ appear to be well conserved in Enterobacteria, some of the σ^S^-independent genes found in our ChIP-seq analysis (*e.g.*, *tomB*, *sdaA*, *bsmA*) appear to be *rpoS*-dependent in *Salmonella* Typhimurium^30^, possibly suggesting more efficient promoter recognition by Eσ^S^ in this bacterium.

Promoter cross-recognition with σ^S^ also seems to extend to the alternative factors σ^E^ and σ^H^ (Tables 1, 2, [Table t2]), in line with previous results showing similar functions of the *rpoE* and *rpoS* regulons and some promoter overlap between the two σ factors *in vitro*^10^,^34^. Indeed, our results confirm a strong interplay between σ^S^ and σ^H^, as the *rpoH* promoter is directly recognized by Eσ^S^ (Table 1), in agreement with its *rpoS*-dependent expression^35^. Our results would be consistent with recent reports showing co-regulation of the *rpoE*, *rpoH* and *rpoS* regulons in response to osmotic stress in enteropathogenic *E. coli* O157:H7^36^, and an extensive analysis of the σ factor network in *E. coli*, showing extensive overlap in promoter recognition by alternative σ’s^33^.

At least 10 of the *rpoS*-dependent genes identified in the ChIP-seq experiments encode small proteins involved in resistance to oxidative stress (*bsmA*, *dps*, *uspB*, *yaiA*, *ychH*, *ydbK*, *ygcG*, *yggE*, *yobF* and *yodD*: Tables 1, 2, [Table t2]), while two more are linked to osmotic stress (*osmB* and *osmE*). Our results would support the notion that, rather than being part of an adaptive response triggered by exposure to specific environmental stresses, the *rpoS* gene activates, in response to reduction in growth rate, a variety of stress-related genes, thus allowing the bacterial cells to “brace themselves” for any stressful conditions that might arise. However, promoter binding by Eσ^S^ does not necessarily translate in increased transcription levels for Eσ^S^-dependent genes, suggesting that, upon binding, Eσ^S^ might be unable to initiate transcription efficiently at some promoters. For the *bsmA* promoter, this hypothesis would fit with the results of *in vitro* promoter interaction studies (Fig. 4) and with our previous results, showing Eσ^S^-dependent transcription of the *bsmA* gene *in vitro*^10^, but not in the bacterial cell. Since *bsmA* is induced in biofilm growth^37^, it is possible that its transcription is repressed in planktonic cells, and triggered during biofilm growth. Thus, our results suggest that Eσ^S^ might be poised at various promoters waiting for additional signals (*e.g.*, leading to removal of a repressor protein) in order to form a complex proficient in transcription initiation.

While stress responses are well known examples of gene functions associated with the *rpoS* regulon, our results suggest direct involvement of σ^S^ in the expression of genes involved in biogenesis and structure of the LPS and outer membrane proteins (Tables 1, 2, [Table t2]). Indeed, changes in cell surface structure and composition are known to take place in stationary phase^38^. According to our ChIP-seq results, in addition to LPS genes, Eσ^S^ also binds to the promoter of *lpp*, encoding Lpp or Braun lipoprotein, which links the outer membrane to peptidoglycan and is the most abundant outer membrane-associated lipoprotein in *E. coli*^21^. Although *lpp* gene expression does not depend on the *rpoS* gene (Fig. 3), a connection of the *rpoS* gene with the function of Braun lipoprotein is further suggested by the identification of two more binding sites for Eσ^S^ upstream of the *erfK* and *ynhG* genes, encoding two of the four alternative transpeptidases that crosslink Lpp to peptidoglycan. Both the *erfK* and *ynhG* genes had already been described as *rpoS*-dependent^15^,^16^. Thus, it appears that, upon entry in the stationary phase of growth, *rpoS* might be required for maintenance of Lpp-transpeptidase activity in the periplasmic space.

Finally, our results point to a direct role of Eσ^S^ in the finely tuned regulation of non-coding RNAs: for instance, Eσ^S^ promotes transcription of *omrA*, but not of the flanking gene, *omrB* (Fig. 5). Both genes encode very similar non-coding RNAs which target the same genes. It appears possible that different dependence on Eσ^S^ by the two promoters might have evolved so to allow differential expression of the OmrA and OmrB non-coding RNAs in response to different signals, with OmrA induced as part of the *rpoS* regulon. The results of mutagenesis at the -12 position of the *omrA* promoter strongly reinforce the notion that the -12C nucleotide can favourably bias transcription initiation by Eσ^S^ at several promoters^39^. Since both the OmrA and OmrB RNAs affect translation of several outer membrane proteins and extracellular structures such as curli and flagella^40^, their selective regulation might mediate the impact of Eσ^S^ on these structures, contributing to a general reorganization of the bacterial cell surface in response to stationary phase.

## Methods

### Strain construction

The *E. coli* MG1655 His_6_::*rpoS* strain (from now on MG1655-*rpoS*_His6_), carrying an *rpoS* gene in which a 6-histidine tag is added to an otherwise wild type allele, was constructed following the genetic procedures described for allele replacement^41^,^42^. Linear DNA fragments containing a kanamycin resistance gene and the *ccdB* gene under the control of a rhamnose inducible promoter were amplified by PCR from the pKD45 plasmid. The first 45 nucleotides of either primer used for amplification (primers rpoS_OF and rpoS_OR, Supplementary Table S1) correspond to the DNA regions immediately upstream and downstream of *rpoS*, targeting the gene for mutagenesis. After PCR amplification, the resulting DNA fragment including the *kanR-ccdB* cassette was used to transform the DY330 strain^42^; the *rpoS* knockout was then P1-transduced into MG1655, selecting for kanamycin resistance. The Δ*rpoS*::*kanR-ccdB* cassette was then replaced by an otherwise wild type *rpoS* sequence to which an additional sequence coding for a 6-histidine tag (6xHis-tag) had been added by PCR amplification, using the rpoS_IF and rpoS_IR primers (Supplementary Table S1). To this aim, DY330 cells carrying the *rpoS* knockout were transformed by electroporation with a linear DNA fragment encoding for the *rpoS*_His6_ gene, carrying the His-tag at the 3` end. Transformant selection was performed on M9 minimal medium agar plates containing 0.2% rhamnose and 0.01% biotin: due to the toxicity of the *ccdB* gene in the presence of rhamnose, only the cells in which an allele replacement has taken place are able to grow on this medium. The *rpoS*_His6_ allele was P1-transduced into MG1655 carrying the *rpoS::kan-ccdB* knockout, again selecting for loss of the *ccdB* gene by plating on M9 minimal medium agar plates containing 0.2% rhamnose and 0.01% biotin. The stability and functionality of the RpoS protein was verified by Western blot and measurement of HPII catalase activity.

### σ^s^_-His6_ immunoprecipitation

For immunoprecipitation of the σ^S^ protein carrying a 6xHis-tag at its C-terminal end (σ^S^-_His6_), the MG1655-*rpoS*_His6_ strain was grown in 50 ml LB medium at 37 °C with vigorous shaking to an OD_600_ = 3.0. In order to enrich the amount of RNA polymerase bound to promoters, cells were treated with rifampicin, which inhibits transcription initiation blocking RNA polymerase at the transcription start site, following the protocol described^43^. To obtain protein-DNA crosslinking, formaldehyde was added at a final concentration of 1% for 5 minutes at room temperature. The crosslinking reaction was stopped by addition of 0.25 M glycine followed by 20 minute incubation at 4 °C with gentle shaking. The cells were washed, resuspended and treated with 100 μg/ml lysozyme for 30 minutes at 37 °C. The lysate was sonicated in order to fragment chromosomal DNA to a size between 100-400bp, and treated with RNaseI (100 μg/ml) for 15 minutes at 37 °C. Cells debris was removed by centrifugation (10 minutes at 10000X*g*). A 250 μl-fraction of the sample was treated with 100 μg/ml Proteinase K and 5 mM CaCl_2_ for two hours at 42 °C, and then at 65 °C overnight, to remove proteins non specifically bound to DNA. DNA was recovered by phenol-chloroform extraction and analyzed on a 2% agarose gel to verify DNA fragmentation. The sample was mixed at a 5:1 (vol:vol) ratio with protein A/G agarose slurry and incubated for 2 h at 4 °C on a rotating wheel to clear the sample and reduce unspecific binding. Subsequently, the agarose beads were separated from the lysate by centrifugation at 10000X*g*. The cleared lysate was then incubated at 4 °C overnight on a rotating wheel with 5 μl of antibody (rabbit polyclonal to 6XHis-tag, ChIP grade, #9108, Abcam, Cambridge, UK). The rest of the procedure was carried out as previously described^44^.

DNA from untreated MG1655-*rpoS*_His6_ was sonicated and 200 μl were taken to be used as a control in sequencing reactions (Input=non-immunoprecipitated DNA). The Input and immunoprecipitated DNA samples were analyzed with the Agilent Bioanalyzer using the High Sensitivity DNA kit (Agilent Technologies). Five IP samples were pooled on the same DNA purification column (minElute, QIAGEN) to reach 5 ng of total DNA, which is the minimum amount for sequencing library preparation. Two pools of IP DNAs were produced. Prior to sequencing libraries construction, quantitative Real Time reverse transcriptase-PCR (qRT-PCR) was carried out to assess the enrichment of the promoter region of the *rpoS*-dependent *dps* gene in the immunoprecipitated samples in comparison to the Input sample. The sequences of the primers used for qRT-PCR are listed in Supplementary Table S1.

### Library preparation and sequencing procedure

Illumina libraries were prepared either from 5 ng of each of the two pools of immunoprecipitated-DNA (RpoS-IP) or from 5 ng of the two control DNA (Input) following the Illumina TruSeq ChIP-seq DNA sample preparation kit; then each library was sequenced in a lane of a single strand 51 bp Illumina run on a GAIIx sequencer. Raw data are publicly available at Sequence Reads Archive under accession number BioProject SRP041323; BioSample SRS595203; Experiment SRX523029; Run1 SRR1265068; Run2 SRR1271103.

### Statistical and bioinformatic data analysis

Raw reads were mapped against the *Escherichia coli* MG1655 genome using Bowtie^45^ with zero mismatches. The resulting BAM files were processed using SAMtools^46^ and BEDTools^47^. The quality of each sequenced sample was checked using cross-correlation analysis implemented in spp R package^48^. ChIP-seq peak calling was performed using CisGenome^12^ by imposing default parameters. Input data (control DNA) was used to model the background noise.

### Determination of *rpoS*-dependent gene expression *in vivo*

For all gene expression experiments, bacterial strains were grown in LB medium to OD600nm = 3.0. For qRT-PCR, RNA was extracted and experiments performed as previously described^49^, using 16S RNA as reference. Primers used in qRT-PCR experiments are listed in Supplementary Table S1. For northern blots, total RNA was extracted using a hot-phenol procedure, so to maintain small RNA molecules. 5 to 20 μg of RNA were separated onto a 6% denaturing acrylamide gel prior to their electro-transfer onto a nylon membrane. As gene specific probes, 5`-Biotinylated oligomers (Supplementary Table S1) were used at 1 nM in combination with 20 pM of the 5S RNA probe as internal control. Saturation and hybridization were performed with the ULTRAhyb^®^-Oligo buffer (Ambion) at 45 °C and signals were detected using a Chemi nucleic acid detect wmodule (Thermo Scientific Pierce). GFP reporter assays were performed as previously described^50^.

### RNA polymerase *in vitro* assays

RNA polymerase reconstitution, gel mobility shift and KMnO_4_ reactivity assays were performed as previously described^32^. ^32^P-labeled DNA was produced by PCR after 5`-phosphorylation of the primer complementary to the coding strand (see Supplementary Table S1) in order to generate linear DNA pieces of about 250 bp, typically encompassing the first 10 codons of the gene and 220 bp of the upstream DNA, including the promoter region. For gel mobility shift assays (GMSA), complexes between reconstituted RNA polymerase (18 to 150 nM) and DNA (1 nM) were allowed to form for 15 min at 37 °C in K- glu100 buffer (40 mM HEPES, pH 8.0, 10 mM magnesium chloride, 100 mM potassium glutamate, 4 mM dithiothreitol (DTT), and 500 *μ*g/ml bovine serum albumin), in a final reaction volume of 10 μl. The reaction mixture was loaded onto a 5% native polyacrylamide gel after addition of 2.5 μl of heparin-supplemented loading buffer^32^ and gel electrophoresis was carried out in 0.5xTBE buffer at 120 V. Experiments were performed at least twice and gave very similar results.

For KMnO_4_ reactivity assays, 50 nM of either form of RNA polymerase (Eσ^S^ and Eσ^70^) were incubated with about 3 nM of labeled promoter DNA for 20 min at 37 °C in K-glu100 buffer without DTT for complex formation. KMnO_4_ was added to a final concentration of 10 mM and the reaction was stopped after 30 seconds by adding 2 mM DTT. Samples were phenol-extracted and precipitated, treated with 1 mM piperidine, resuspended in pure formamide blue before being loaded onto a 7% polyacrylamide denaturing gel. A DNA ladder was generated for each labeled DNA fragment by partial G/A sequencing using formic acid and piperidine.

### Other methods

Determination of HPII catalase activity and Western blot experiments were carried out as previously described^51^,^52^. Mutagenesis of the *omrA* promoter was carried out by generation of PCR products with mutagenic primers carried the desired substitutions, as previously described^32^.

## Additional Information

**How to cite this article**: Peano, C. *et al.* Characterization of the *Escherichia coli* σ^S^ core regulon by Chromatin Immunoprecipitation-sequencing (ChIP-seq) analysis.. *Sci. Rep.* **5**, 10469; doi: 10.1038/srep10469 (2015).

## Supplementary Material

Supplementary Information

## Figures and Tables

**Table 1:** Location of putative Eσ^S^ binding sites attributable to a specific promoter region.

**peak start**	**peak end**	downstream gene*	**chromosome strand**	**experimentally validated TSS located inside peak**	**Gene function**	**References showing gene regulation by σ**^**S**^ **or by other alternative σ factors**
63400	63538	*hepA*	-		RNA-polymerase associated ATPase	13 (**σ**^**H**^ )
106436	106616	***lpxC***	+	106530	UDP-3-O-acyl-N-acetylglucosamine deacetylase (lipid A biosynthesis)	18
262040	262202	*thrW*	+		threonyl-tRNA	
392250	392349	***insEF-2***	-		IS-3 transposase	17
406100	406199	***yaiA***	+		unknown, oxidative stress	16
437329	437469	***yajO***	-		putative NAD(P)H-dependent xylose reductase	16
479920	480115	*tomB*	-		antitoxin in tomB/hha T/A system	
574850	575099	*insH-2*	-		IS-5 transposase	
837550	837849	*ybiI*	-		unknown	
848050	848349	*dps*	-	848173	stationary phase nucleoid component/ferritin	14,15,16
1215900	1216399	***ymgC***	+		involved in biofilm formation	15
1219400	1219949	*ycgH*			pseudogene- autotransporter	
1236420	1236526	***ycgB***	-	1236508	unknown	15,16
1341304	1341480	***osmB***	-	1341393	osmotically inducible lipoprotein	15,53,54
1430250	1430549	*tfaR*	+		Rac prophage tail fiber assembly protein, induced in biofilms	
1509526	1509697	***ydcS***	+	1509623	polyamine transporter	15,16,55
1524000	1524199	***ansP***	-	1524035 / 1524044	arginine transporter	14,18
1608700	1608949	*uxaB*	-	1608744	galacturonate degradation	
1687744	1687907	***ydgA***	+	1687818	unknown, involved in swarming motility	16,18
1755350	1755499	*lpp*	+	1755407	Braun lipoprotein	
1756820	1756885	***ynhG***	-		transpeptidase, associated to Lpp	15,16
1894663	1894896	*sdaA*	+	1894833	serine deaminase	13 (**σ**^**H**^)
1905547	1905784	*yobF*	-	1905641	stress response protein	
1920033	1920203	*yebW*	+		unknown	
1921150	1921299	***ryeB***	-		small RNA, antisense of small RNA *ryeA*	10,29
2026384	2026505	***yodC***	-		unknown	15,16
2061261	2061484	***erfK***	-		transpeptidase, associated to Lpp	16
2103850	2104199	*wbbI*	-		β-1,6-galactofuranosyl-transferase, LPS O-antigen	
2104550	2105599	*wbbH*	-		LPS O-antigen polymerase	
2190800	2190949	*yehE*	-		unknown	
2225279	2225390	***yohF***	-		predicted acetoin dehydrogenase	16
2468677	2468882	*tfaS*	+		CPS-53 prophage tail protein	
2663364	2663501	***csiE***	+	2663423	stationary phase inducible gene	15,16,56
2734910	2735081	***raiA***	+		ribosome inhibitor, stationary phase-dependent	13 (**σ**^**H**^); 19
2753502	2753707	*ssrA*	+	2753608	tmRNA	
2758300	2758999	***yfjJ***	+		CP4-57 prophage protein	^17^
2797100	2797249	*alaE*	+		alanine exporter	
2817227	2817395	***csrA***	-	2817295	RNA-binding protein, translational regulator	18,57
2924252	2924370	***ygdH***	+		unknown	19
2974153	2974278	***omrA***	-	2974211	small regulatory RNA	30
2991100	2992299	*ygeI*	+		unknown	
3054792	3054952	*sibC*	+	3054873	small regulatory RNA	
3058600	3058749	*scpA*	+		methyl-malonyl-CoA mutase	
3066050	3066149	***yggE***	-	3066148	unknown, oxidative stress	14,16
3235233	3235381	*ygjR*	+	3235304	predicted dehydrogenase	
3598950	3599099	***rpoH***	-		alternative sigma factor (sigma32)	35
3637750	3637949	***uspB***	-	3637871	universal stress protein B	16,18,58
3706750	3706999	*proK*	-		prolinyl-tRNA	
4361287	4361432	***yjdC***	-	4361353	putative transcriptional regulator	16
4437000	4437349	***ytfJ***	-	4437309	unknown, periplasmic protein	19; 59 (**σ**^**E**^)

^*^Genes for which regulation by σ^S^ has already been shown (see last column) are indicated in boldface type; genes with promoter DNA regions that were studied *in vitro* are underlined.

**Table 2 t2:** Location of putative Eσ^S^ binding sites in intergenic regions between divergent genes.

**peak start**	**peak end**	nearest gene* **(- strand)**	**Gene function**	**experimentally validated TSS inside the peak**	nearest gene* **(+ strand)**	**Gene function**	**References showing gene regulation by σ**^**S**^ **or by other alternative σ factors**
1257750	1258199	*pth*	peptidyl-tRNA hydrolase	1257765 (*pth*) 1257961 (*ychH*)	***ychH***	unknown, oxidative stress	19
1288250	1288399	*ychJ*	unknown	1288400 (*ychJ*) 1288329(*rssA*)	***rssA***	unknown	16
1438800	1439049	*ydbK*	pyruvate flavodoxin oxidoreductase, involved in oxidative stress	1439053 (*ydbJ*)	*ydbJ*	unknown	
1488650	1488949	**(*gapC_1*)**	glyceraldehyde 3-phosphate dehydrogenase (pseudogene)		*cybB*	cytochrome b561	18,19
1820250	1820349	***osmE***	osmotically inducible lipoprotein	1820307(*osmE*) 1820326 (*nadE*)	*nadE*	NAD synthetase, NH_3_-dependent	15,16
2022850	2023149	***dsrB***	unknown		***yodD***	involved in oxidative and acid stress	15,16,18
2493450	2493549	*yfdY*	biofilm-dependent membrane protein		***lpxP***	palmitoleoyl acyltransferase (LPS biosynthesis)	60 (**σ**^**E**^)
2627100	2627399	*yfgF*	c-di-GMP phosphodiesterase	2627275 (*yfgG*)	*yfgG*	unknown	
2903350	2903649	*queE*	conserved protein		***ygcG***	small protein involved in cell envelope stress	17
3851100	3851399	*istR-1/istR-2*	regulatory small RNA for *tisB*	3851215-3851280 (*istR*) 3851360 (*tisB*)	tisB	toxic peptide	
4124850	4125049	*priA*	DNA replication restart factor	4124931 (*rpmE*)	*rpmE*	L31 ribosomal protein	13 (**σ**^**H**^)
4414650	4414899	***bsmA***	biofilm-dependent protein involved in oxidative stress		*yjfP*	esterase	10,19
4434400	4434749	*cpdB*	2'3' cyclic nucleotide phosphodiesterase and nucleotidase	4434652 (*cpdB*)	*cysQ*	adenosine 3'-5' bisphosphate (PAP) nucleotidase	19

^*^Genes for which regulation by σ^S^ has already been shown (see last column) are indicated in boldface type; genes with promoter DNA regions that were studied *in vitro* are underlined.
