# Supplementary material for: Characterization of the Escherichia coli σS core regulon by Chromatin Immunoprecipitation-sequencing (ChIP-seq) analysis
Source: Sci Rep. 2015 May 28;5:10469. doi: 10.1038/srep10469 (PMC4447067; doi:10.1038/srep10469)
Supplement: Supplementary Information [file srep10469-s1.pdf]

Supplementary Materials

Characterization of the *Escherichia coli* σ^S core regulon by Chromatin Immunoprecipitation-sequencing (ChIP-seq) analysis.

Clelia Peano[1+], Johannes Wolf[2+], Julien Demol[3,4], Elio Rossi[5], Luca Petiti[1], Gianluca De Bellis[1], Johannes Geiselmann[3,4], Thomas Egli[2], Stephan Lacour[3,4*] and Paolo Landini[5*]

[1] Institute of Biomedical Technologies, National Research Council (ITB-CNR), Segrate (MI), Italy

[2] EAWAG, Swiss Federal Institute for Environmental Science and Technology, Dübendorf, Switzerland

[3] Lab. Adaptation et Pathogénie des Micro-organismes (LAPM), Univ. Grenoble Alpes, F-38000 Grenoble, France.

[4] UMR 5163, Centre National de Recherche Scientifique (CNRS), Grenoble, France

[5] Department of Biosciences, Università degli Studi di Milano, Milan, Italy

* To whom correspondence should be addressed:

*Stephan Lacour
Tel. +33 4 76 63 74 89
Fax. +33 4 76 63 74 97
stephan.lacour@ujf-grenoble.fr

*Paolo Landini
Tel. +39-02-50315028
Fax +39-02-50315044
paolo.landini@unimi.it

[+] C.P. and J.W. contributed equally to this work

Table S1. Sequence and use of DNA primers used in the present study.

Primer name	Sequence	Utilization
rpoS_OF	TTTGCTTGAATGTTCCGTCAAGGGATCACGGGT-AGGAGCCACCTTTCAGAAGAACTCGTCAAGAAGG	Knock out of *rpoS*
rpoS_OR	AAAGGCCAGCCTCGCTTGAGACTGGCCTTTCTG-ACAGATGCTTACCGTCATCGCCATTAATTCACTG	Knock out of *rpoS*
rpoS_IF	TTTGCTTGAATGTTCCGTCAAGGGATCACGGGT-AGGAGCCACCTTATGAGTCAGAATACGCTGAAA	Knock in replacement of *rpoS*
rpoS_IR	AAAGGCCAGCCTCGCTTGAGACTGGCCTTTCTG-ACAGATGCTTACTTAGTGATGGTGATGGTGATGC-TCGCGGAACAGCGCTTCG	Knock in replacement of *rpoS*
rpoS_F	AACCAGTTCAACACGCTTGC	PCR verification of *rpoS* locus
rpoS_R	GTGTTAACGACCATTCTCGG	PCR verification of *rpoS* locus
dps_F	GCCAGAATAGCGGAACACAT	qRT-PCR (ChIP enrichment)
dps_R	GCGGGTATAAAGCAGATTGG	qRT-PCR (ChIP enrichment)
rpoB_F	GCGATGGCGTAGAAAAAGAC	qRT-PCR (ChIP enrichment)
rpoB_R	CTACCAGCACAGCACGGATA	qRT-PCR (ChIP enrichment)
yeeJ_F	GACGAACGAACAGGGTAAGG	qRT-PCR (ChIP enrichment)
yeeJ_R	TTGATGTGCTAAGCGTGGAG	qRT-PCR (ChIP enrichment)
dps_F	GGATGGCTTCCGCACCG	qRT-PCR
dps_R	CTTGAGTGGTCCCCAGAG	qRT-PCR
ycgB_F	TCCGAACCACAAGAAAACCT	qRT-PCR
ycgB_R	GGCTCACCTTACGCACAATAC	qRT-PCR
lpp_F	AGCTGAGCAACGACGTGAAC	qRT-PCR
lpp_R	GTAGCCATGTTGTCCAGACG	qRT-PCR
ssrA_F	ATCAAGAGAGGTCAAACCCAAA	qRT-PCR
ssrA_R	GGACGGACACGCCACTAAC	qRT-PCR
uxaB_F	ATTCAGGGACCGAAATCCTT	qRT-PCR
uxaB_R	ATTGCCACTTTGCGTTCTTT	qRT-PCR
ybiI_F	CCGTCAACGAACAGATCAAC	qRT-PCR
ybiI_R	CCGCACTCTTCACATTCATC	qRT-PCR
ydbK_F	AAAGTTGGCGTGCTGAAAGT	qRT-PCR
ydbK_R	GGGTTCTTTGGTTCTGTCCA	qRT-PCR
ygjR_F	CTGTTTACCTCGCTGGAAGC	qRT-PCR
ygjR_R	CGGTTTCTCGCAAATCACAT	qRT-PCR
16S_F	TGTCGTCAGCTCGTGTCGTGA	qRT-PCR
16S_R	ATCCCCACCTTCCTCCGGT	qRT-PCR
ybiI_ fwd	TATTCAGTCGCTGAAAAGC	*In vitro* experiments with labelled DNA

ybiI_ rev	AATTGTACTGTTGATCTGTTCG	*In vitro* experiments with labelled DNA
ydbK_fwd	CGCATTTTTACTTTTTTATGGC	*In vitro* experiments with labelled DNA
ydbK_rev	GATGGCGATAACTTCACTGG	*In vitro* experiments with labelled DNA
omrA_Fwd	TATCTCGAGGCCGGTCATCAATCTGTAAC	Construction of the *omrA::GFP* transcriptional fusion
omrA_rev	TATGAATTCCCTCTGGGATCTTGATTGTG	Construction of the *omrA::GFP* trasncriptional fusion
omrB_Fwd	TATCTCGAGTCAGTGTTACGGAAAACGCC	Construction of the *omrB::GFP* transcriptional fusion
omrB_rev	TATGAATTCCCTCTGGGATCACCACTTTA	Construction of the *omrB::GFP* transcriptional fusion
omrA_mut_rev	TATGAATTCCCTCTGGGATCTTGATTGT**<u>A</u>**GTCTGC	Construction of the mutant omrA::GFP transcriptional fusion*
[Btn]-omrA	TTGGTGCAAGAGACAGGGTACGAAG	Northern blot hybridization**
[Btn]-sibC	CATCATACTGATTGTACTGTTACTC	Northern blot hybridization**
[Btn]-ibsC	GAGTAACAGTACAATCAGTATGATG	Northern blot hybridization**
[Btn]-ryeA	AGAGCCATTTCCCTGGACCGAATAC	Northern blot hybridization**
[Btn]-ryeB	CCTTGCCCTTTAAGAATAGATGACG	Northern blot hybridization**
[Btn] 5S RNA	CGGCGCTACGGCGTTTCACTTCTG	Northern blot hybridization**

*: the C>T substitution in the omrA -10 element (G>A in the reverse complement strand) is indicated in bold and underlined

**:[Btn] indicates a 5'-biotinylation of the primer for utilization in northern blot experiments

Table S2. Location of intragenic peaks identified in ChIP-seq analysis.

peak start	peak end	peak length	inside gene	chromosome strand	Gene function	Distance (bp) from closest gene start/ (gene)
130200	130349	149	yacH	-	putative membrane protein	911 (yacH)
390600	390799	199	yaiT	+	putative outer membrane protein	1125 (yaiT)
579200	579449	249	ybcW	+	DLP2 prophage, predicted protein	97 (ybcW)
783131	783309	178	zitB	+	zinc metal transporter	737 (zitB)
951282	951339	57	pflB	-	pyruvate-formate lyase	979 (pflA)
1260424	1260539	115	prs	-	ribose phosphate diphosphokinase	398 (dauA)
2190700	2190799	99	yehE	-	predicted protein	19 (yehE)
2480950	2481249	299	emrK	-	Multidrug efflux transport system	112 (emrK)
2987500	2990649	3149	yqeL/yqeK/ygeF ygeG/ygeH	both	predicted proteins, chaperone, putative transcription regulator	N/A
2992550	2992699	149	ygeK	-	transcription regulator, NarL family	226 (ygeK)
3362550	3362699	149	yhcD	+	predicted outer membrane protein	510 (ychE1)
3471311	3471368	57	fusA	-	translation elongation factor G	168 (fusA)
3472750	3472899	149	tusB	-	sulphur transfer protein complex	88 (tusB)
4366056	4366171	115	aspA	-	aspartate-ammonia lyase	179 (aspA)
4533950	4534049	99	yjhR (pseudogene)	+	Rac prophage tail fiber assembly protein, induced in biofilms	912 (yjhR)

Supplementary Table S3. **Sequence of experimentally determined -10 promoter regions lying within ChIP-seq peaks.**

Promoter name	TSS location (coordinates)	Promoter sequence (-20 to +1 region)
**σ^S-dependent genes**		
ansP (P1)	1524035	GTTCATGG**<u>CAACTT</u>**ATAT**GA**
ansP (P2)	1524044	TGAATAAA**<u>CATTGT</u>**TCATGG
cpdB	4434652	AATTGTGG**<u>CATTCT</u>**TCACTG
csiE	2663423	TCCTTGAG**<u>CAAACT</u>**TTAGCT
csrA	2817295	ATATCGG**C<u>TAAACT</u>**TAGGTT
dps	848173	GCCGGTG**C<u>TATACT</u>**TAATCT
omrA	2974211	GTGCAGA**C<u>CACAAT</u>**CAAGAT
osmB	1341393	TTCTTAG**C<u>TATTAT</u>**AGTTAT
osmE	1820307	ATGTATT**C<u>CAGGCT</u>**TATCTA
rssA	1288329	ACATAGG**C<u>TATACT</u>**CGACAG
sibC	3054873	CTTTGAC**C<u>TAATTT</u>**AGTGAG
uspB	3637871	TGATCTC**C<u>TACACT</u>**ATCT**AT**
ybiI	837707	GGATTAG**C<u>TACACT</u>**TAACT**G**
ycgB	1236508	CAGGTGTT**C<u>TATGCT</u>**TGAAA
ychH	1257961	GTGTTGT**C<u>TATACT</u>**TTACAC
ydbK	1438870	CCATTTG**C<u>TAAAAT</u>**TGAAAG
ydcS	1509623	CGCCGGT**C<u>TATGGT</u>**TAAACA
ydgA	1687818	TCTCTTG**C<u>TAAGCT</u>**TATTAA
yggE	3066148	AGAGGCG**C<u>TAAGCT</u>**TGCCTC
yjdC	4361353	CCGTGAA**C<u>GACACT</u>**TGCAGT
ytfJ	4437309	CCAAGTGG**<u>TCGGAT</u>**CACC**TG**
**σ^S-independent genes**		
alaE	2796688	CAAGATTA**TATATT**AGCCAT
lpp	1755407	ACTTTGTG**TAATAC**TTGTAA
lpxC	106530	ATGTATAG**<u>TACACT</u>**TCGGTT

sdaA	1894833	AATCATCG**CAATAT**TAGTTA
ssrA	2753608	AAATCTGG**TATACT**TACCTT
uxaB	1608744	AAGTTTG**CTAACCT**GTCGTA
ygjR	3235304	AGGCGTG**CTACATT**GACGAC
yobF	1905641	GTATGTGA**TAACAG**ATTTC**G**
ytfJ	4437309	CCAAGTGG**TCGGAT**CACC**TG**

Supplementary Table S4. Sequence of experimentally determined -35 promoter regions lying within ChIP-seq peaks

Promoter name	TSS location (coordinates)	Promoter sequence (-35 region)
**σ^S-dependent genes**		
ansP (P1)	1524035	GGATGAATAA
ansP (P2)	1524044	AACTATCATC
cpdB	4434652	CATTTTCAAA
csiE	2663423	CATTCCTTTC
csrA	2817295	GTCAGGTTGA
dps	848173	AATAGCGGAA
omrA	2974211	CGCTGGCGAA
osmB	1341393	ATATTTCACC
osmE	1820307	GCTTGAAAAA
rssA	1288329	GACACATAAG
sibC	3054873	GATTGACATC
uspB	3637871	TCTGGAAAAA
ybiI	837707	TGCCCGAGTT
ycgB	1236508	CGGCGACGCG
ychH	1257961	CGCACATAAC
ydbK	1438870	CGAAAATGCA
ydcS	1509623	CGAAGGTGTG
ydgA	1687818	TACTGAAAAA
yggE	3066148	TGATGGAAAA
yjdC	4361353	TTTTGGCGGA
**σ^S-independent genes**		
alaE		AACTCTCAGA
lpp	1755407	TATTCTCAAC
lpxC	106530	GCTAAACTGG

sdaA	1894833	AAAACACTAC
ssrA	2753608	GCTGGTCATG
uxaB	1608744	GGTTCGGCGT
ygjR	3235304	CGTCGAAGAT
yobF	1905641	GCCTGGAACA
ytfJ	4437309	GATTAAACTC

Supplementary Figure-S1 (Landini)

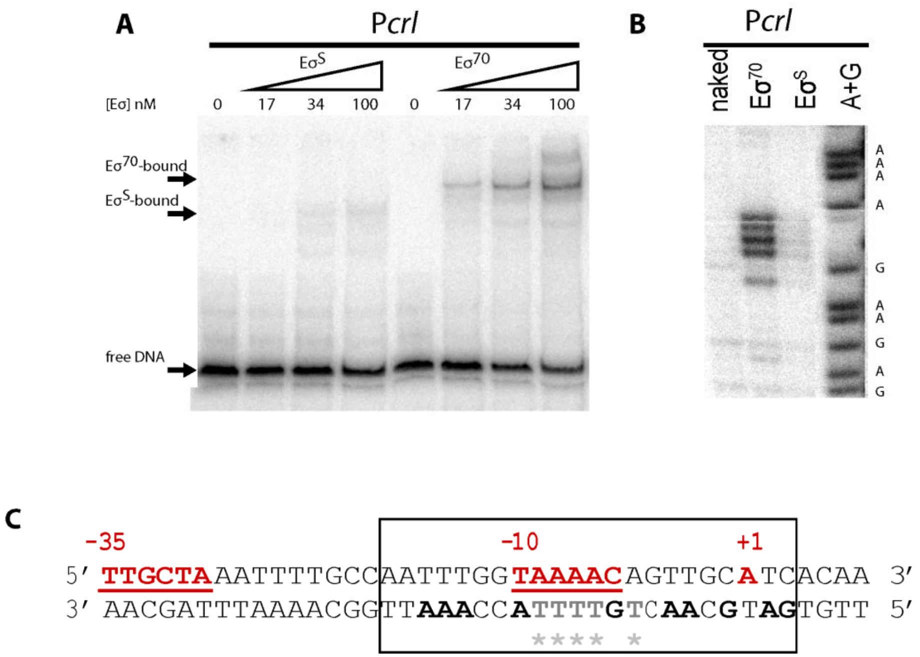

Supplementary Figure-S1. Eσ^{70} and Eσ^{S} interaction with the *crl* promoter *in vitro*. A. Gel retardation assays performed in K-glutamate buffer with heparin challenge. **B.** $KMnO_4$ reactvity assays: both Eσ^{S} and Eσ^{70} forms of RNA polymerase were tested at 50 nM.; a G+A sequencing reaction of the *crl* promoter fragment (template strand) was used as molecular weight marker (residues marked in bold in panel C). **C.** Sequence of *crl* promoter. Promoter elements are indicated in red and the -35 and -10 sequences are underlined.

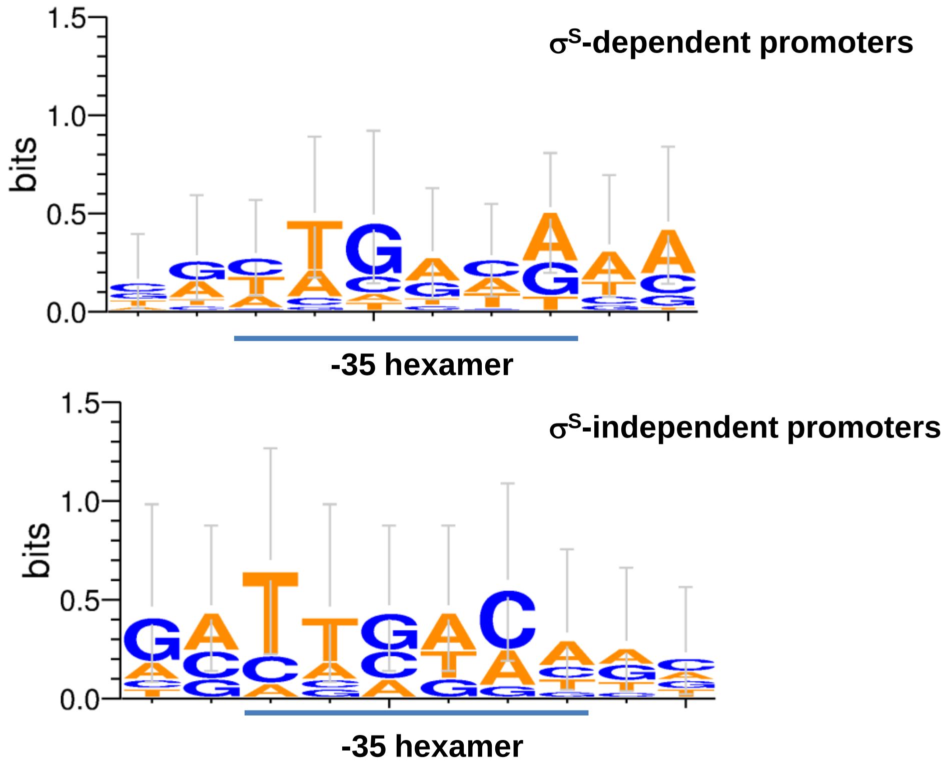

Supplementary Figure S2. Promoter sequence alignment. Weblogo 3 (http://weblogo.threeplusone.com/) representation of the sequence alignments for experimentally identified promoters located within Eσ^S binding sites. -35 regions of either σ^S-dependent (top panel) or σ^S-independent genes (bottom panel) were aligned based on the first nucleotide of the putative -35 hexamer. -35 sequences are reported in Table S4.
